# Apocynin suppression of NADPH oxidase reverses the aging process in mesenchymal stem cells to promote osteogenesis and increase bone mass

**DOI:** 10.1038/srep18572

**Published:** 2015-12-21

**Authors:** Jinlong Sun, Leiguo Ming, Fengqing Shang, Lijuan Shen, Jihua Chen, Yan Jin

**Affiliations:** 1 State Key Laboratory of Military Stomatology, Center for Tissue Engineering, School of Stomatology, Fourth Military Medical University, Xi’an, Shaanxi 710032, China; 2 Research and Development Center for Tissue Engineering, Fourth Military Medical University, Xi’an, Shaanxi 710032, China; 3 State Key Laboratory of Military Stomatology, Department of Oral Histology and Pathology, School of Stomatology, Fourth Military Medical University, Xi’an, Shaanxi 710032, China; 4 State Key Laboratory of Military Stomatology, Department of Prosthodontics, School of Stomatology, Fourth Military Medical University, Xi’ an, Shaanxi 710032, PR China; 5 Institute for Tissue Engineering and Regenerative Medicine Research of Xi’an, Xi’an, Shaanxi, 710032, China

## Abstract

Because of the reduced potential for osteogenesis in aging bone marrow stromal cells, the balance of bone metabolism becomes disrupted, leading to various bone diseases. An increase in reactive oxygen species has been determined to be one of the key factors that accelerates the aging process in BMSCs. In these cells, increased expression of NADPH oxidases is the major source of ROS. In the current study, we suppressed the expression of NOX using apocynin, an effective antioxidant and free radical scavenger, and the results showed that aging BMSCs exhibited an enhanced potential for osteogenesis. The expression of potential key targets influencing this reversal was evaluated using qRT-PCR, and the expression of p53 was shown to be reduced with the suppression of NOX. We speculate that this may be one of the major reasons for the reversal of the aging process. We also examined the effect of apocynin *in vivo*, and the results showed that in SAMP6 mice, bone mineral density and total bone volume were increased after 3 months of apocynin treatment. In conclusion, our results demonstrate that in aging BMSCs, suppression of NADPH oxidase by apocynin partially reverses the aging process and enhances osteogenic potential.

Bone marrow stromal cells (BMSCs) play a key role in maintaining the balance of bone metabolism^1^,^2^,^3^. The differentiation of BMSCs into osteoblasts is the major source of bone mass^1^,^4^. During the aging process, BMSCs become senescent, and their potential for osteogenesis is reduced^5^, leading to an imbalance in bone metabolism and resulting in various bone diseases related to aging^6^. Therefore, it is important to regulate the aging process in BMSCs to maintain the balance of bone metabolism.

There are various factors that influence the cell aging process, and oxidative stress has been shown to be one of the key factors that affects aging^7^,^8^. Since Denham Harman first proposed the role of oxidative stress in aging, numerous reports have examined the relationship between oxidative stress and aging^7^,^9^. Whether oxidative stress affects aging has been a controversial topic for years. However, reactive oxygen species (ROS) are known to be one of the major factors involved in the aging process in BMSCs and are involved in various intracellular signalling pathways^10^. The continuous damage caused to cellular macromolecules by ROS leads to a progressive decline in the function of cellular processes, finally resulting in aging of the organism^11^,^12^. It has been reported that the aging of bone tissue is a unique process due to its sclerous characteristic^13^. Numerous studies focused on the functional equilibrium between osteoblasts and osteoclasts have indicated that disruption in this balance leads to bone metabolic disturbance and bone aging^14^. However, one particularly important factor in bone aging, the aging of BMSCs, has received less focus in recent studies. We believe that with the senility of an organism, the BMSC aging process directly affects bone aging. Hence, elucidating the role of oxidative stress in the aging process in BMSCs is important for understanding the process of bone aging.

In this study, an inhibitor of NADPH oxidase (NOX), apocynin, was chosen to perform cell treatments. Apocynin has been shown to be an effective down-regulator of intracellular ROS in numerous studies^15^,^16^. In recent years, many reports have indicated that apocynin does not inhibit the activity of NADPH oxidase or even up-regulate the expression of ROS in many cell models^17^,^18^,^19^. Here, we report for the first time that in a senescent cell model and in mesenchymal stem cells, apocynin suppresses NADPH oxidase and reduces intracellular ROS. Based on these findings, the data showed that the aging process in BMSCs was partially reversed and the osteogenesis potential of the BMSCs was enhanced. We also determined that p53 plays a pivotal role in the series of molecular reactions that occur downstream of ROS. Furthermore, we evaluated the effect of apocynin in a mouse model, and the results demonstrated that the potential for osteogenesis was increased, while the ROS level was decreased.

## Results

### Optimal concentration of apocynin in BMSCs

**Figure 1:**
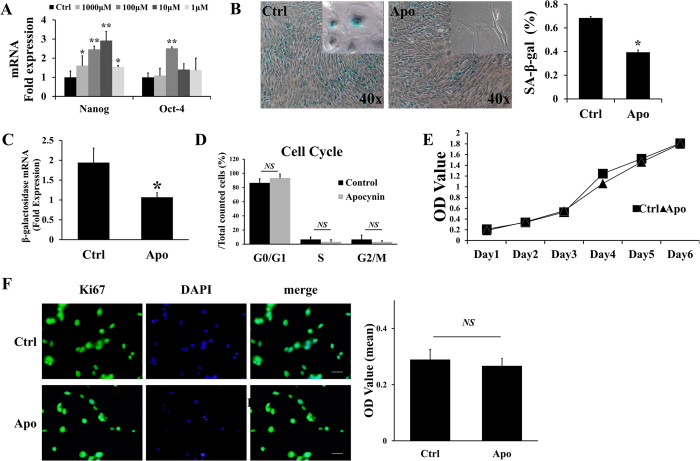
RT-PCR was used to evaluate the effect of different concentrations of apocynin on the expression of nanog and oct-4 (A). Apocynin treatment (100 μM) of aging BMSCs significantly reduced the number of SA-β–gal-positive cells, by 42.5% compared with the negative control (**B**). The RT-PCR results showed that the reduction of β–gal expression occurred at the mRNA level (**C**). There appeared to be no change in the cell cycle (**D**) or proliferation under apocynin treatment at 100 μM compared with the negative control (**E**,**F**).

Aging BMSCs were isolated from 22-month-old SD rats and were used to determine the optimal concentration of apocynin. BMSCs isolated from 4-week-old SD rats were employed as a control. β–galactosidase staining was performed to identify aging BMSCs. The results showed that there was a higher percentage of SA-β–gal-positive (green-stained) cells among the BMSCs isolated from the 22-month-old SD rats than in the control cells isolated from the 4-week-old SD rats (50.55 ± 1.22% vs. 3.10 ± 1.05%) (Fig. S1A). This result indicated that the BMSCs isolated from the 22-month-old SD rats met the standard of aging cells and could be used as aging BMSCs in the following experiments. The main purpose of the next set of experiments was to determine the effect of apocynin at different concentrations on aging BMSCs. We choose Nanog and Oct-4 as the targets of this concentration screen, and their expression levels were determined via qRT-PCR. Nanog and Oct-4 are “stemness markers” that play important roles in self-renewal and maintaining the potential of the stem cells to differentiate^20^,^21^. The qRT-PCR results showed that 100 μM apocynin caused significant increases in Nanog and Oct-4 levels, of 2.4 fold and 2.5 fold, respectively, compared with the control group treated with the drug carrier solvent DMSO (Fig. 1A). The results of MTT assays also confirmed that 100 μM apocynin showed no cytotoxicity in the BMSCs (Fig. S1B). Based on these findings, we used apocynin at a 100 μM concentration in the following experiments.

### Aging-related changes of BMSCs treated with apocynin

The results showed that the expression levels of the “stemness markers” Nanog and Oct-4 were increased under apocynin (100 μM) treatment in the aging BMSCs. Therefore, in the next experiment, we investigated whether the aging process in BMSCs was reversed or partially reversed by apocynin. We performed SA-β-gal staining in aging BMSCs treated with apocynin (100 μM); DMSO was used as a negative control. A total of 5 × 10^4^ aging BMSCs were seeded into each well of a 12-well plate. After 12 h, the cells were treated with either apocynin (100 μM) or DMSO. Staining was then conducted after incubation for 24 h. The results showed that the percentage of SA-β–gal-positive (green-stained) cells was decreased by 42.5% under apocynin treatment compared with the negative control (Fig. 1B). We also determined β–gal mRNA levels using a qRT-PCR assay and found that the expression of β–gal was decreased by 55.0% in cells treated with apocynin (Fig. 1C). Next, we investigated whether apocynin at a 100 μM concentration affects the cell cycle and proliferation. The results of flow cytometry and MTT assays showed that apocynin at 100 μM did not cause any changes in the aging BMSCs (Fig. 1D,E). Cell immunofluorescence was also used to examine the level of the marker of proliferation, Ki67. And results showed that apocynin did not change the expression of Ki67 (Fig. 1F).

### Modification of senescence-related markers

**Figure 2:**
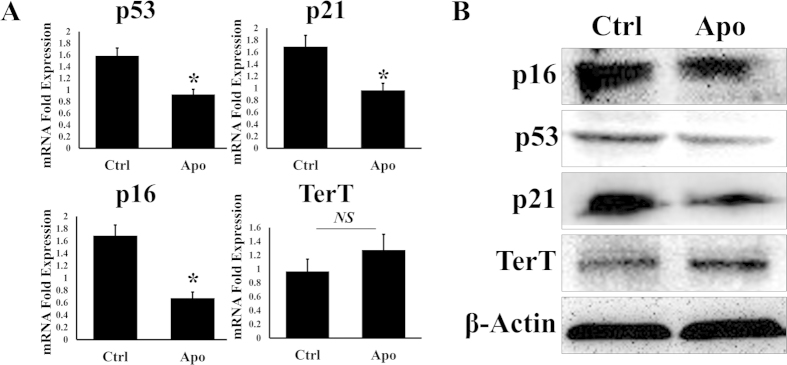
The expression levels of p53, p21 and p16 were decreased at both the mRNA level (**A**) and protein level (**B**) with apocynin treatment (100 μM). The expression of TERT was increased (**B**) with apocynin treatment (100 μM) in protein level, while in mRNA level, RT-PCR results showed no statistical differences (p > 0.05).

The results of SA-β-gal staining assays showed that treatment with apocynin at 100 μM partially reversed the aging process in BMSCs. Next, we investigated various targets related to aging using a qRT-PCR assay and expected to find the main factors that influence the reversal of the aging process. The results showed that 100 μM apocynin increased the expression of sox-2 and klf-4 by 82.4% and 38.7%, respectively, compared with the negative control group (Fig. S2). Besides, expression of c-myc was also increased in mRNA level (Fig. S2).The protein and mRNA expression levels of p53, p21 and p16 were all decreased compared with the expression levels in the negative control (Fig. 2A,B). The protein expression level of telomerase reverse transcriptase (TERT) was increased, but the qRT-PCR results showed that there was no significant difference in the TERT mRNA expression level compared with the negative control (Fig. 2A,B). We also determined SIRT1 and SIRT2 expression levels using qRT-PCR, and the results showed that there was no difference between the two groups. Additionally, the expression levels of AP2 (adipocyte fatty-acid-binding protein) were higher compared with the negative control (Fig. S2).

### Changes of p53 expression is related to ROS level

The results showed that there was a significant change in the p53 expression level during the apocynin-induced reversal of the aging process in BMSCs. We speculated that p53 plays an important role in altering the aging of BMSCs. We also speculated that apocynin down-regulated the activity of NADPH oxidase in the aging BMSCs, decreasing the level of intracellular ROS, resulting in a series of changes in downstream targets, particularly p53 expression. The suppression of p53 activated downstream targets and resulted in aging-associated changes (Fig. S3).

**Figure 3:**
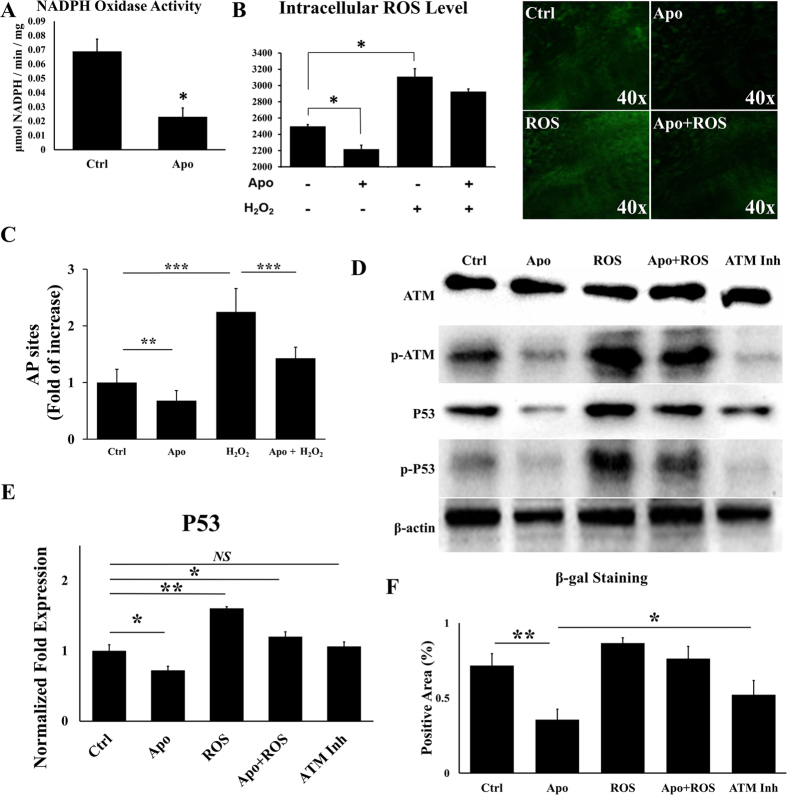
Apocynin (100 μM) reduced the expression of NADPH oxidase by 66.5% compared with the negative control (A). Apocynin (100 μM) also reduced the level of intracellular ROS, while H_2_O_2_ (50 μM) enhanced the level of ROS (**B**). Cells simultaneously treated with both apocynin and H_2_O_2_ exhibited a slight increase compared with the negative control group (**B**). DNA damage was examined. Apocynin/ROS treatment up/down regulated the numbers of AP sites respectively (**C**). The RT-PCR and western blot results showed that the expression of p53 was positively associated with the level of ROS through ROS-DNA damage-ATM-p53 pathway (**D**,**E**). SA-β–gal-positive cells were increased/reduced by the treatment of H_2_O_2_ and apocynin respectively (**F**).

To determine the validity of our proposed molecular mechanism, we investigated the activity of NADPH oxidase in aging BMSCs with or without apocynin (100 μM) treatment. The results revealed that apocynin reduced the expression level of NADPH oxidase by 66.5% compared with the negative control (Fig. 3A). Next, we investigated whether apocynin reduces the level of intracellular ROS. We set up a group treated with H_2_O_2_ (50 μM) and a group treated with both H_2_O_2_ (50 μM) and apocynin (100 μM) as a positive control. Flow cytometry analysis showed that apocynin reduced the level of intracellular ROS by 11.2%, while H_2_O_2_ (50 μM) increased the level by 24.5% compared with the control group treated only with DMSO. The aging BMSCs treated simultaneously with apocynin and H_2_O_2_ displayed a 17.2% increase in the ROS level, which was higher than in the negative control and lower than in the H_2_O_2_-treated group (Fig. 3B). Under a laser scanning confocal microscope (LSCM), we observed results that were consistent with the results from the flow cytometry analysis (Fig. 3B). Then the DNA damage was examined. Results showed that with the treatment of apocynin, numbers of AP sites was obviously reduced, while the treatment of H_2_O_2_ increased the numbers of AP sites (Fig. 3C).

Next, we investigated whether the expression of p53 is correlated with the level of intracellular ROS. We detected the expression of p53 through qRT-PCR. And expression of p53, ATM and their phosphorylation were also detected by western blot. The western blot results showed that the expression of phosphorylated ATM was positively associated with the level of ROS and DNA damage. Expression of p53 and its phosphorylation was up/down regulated by the treatment of apocynin/H_2_O_2_ respectively. The ATM inhibitor KU-55933 was also used to treat the cells (20 μM). Results showed that after the treatment, phosphorylation of ATM and p53 were both inhibited (Fig. 3D). The results of qRT-PCR analysis also support the conclusion (Fig. 3E) that the reduced intracellular ROS level resulting from apocynin (100 μM) treatment suppressed the expression of p53, leading to a series of downstream changes associated with the change in the aging process in BMSCs. Here we examined the expression of β-galactosidase. Results showed that with the increase of ROS, cells expression more β-galactosidase. Apocynin and KU-55933 cut down the expression of β-galactosidase by 50.29% and 27.19% respectively (Fig. 3F).

### Osteogenic potential of BMSCs was promoted by apocynin

**Figure 4:**
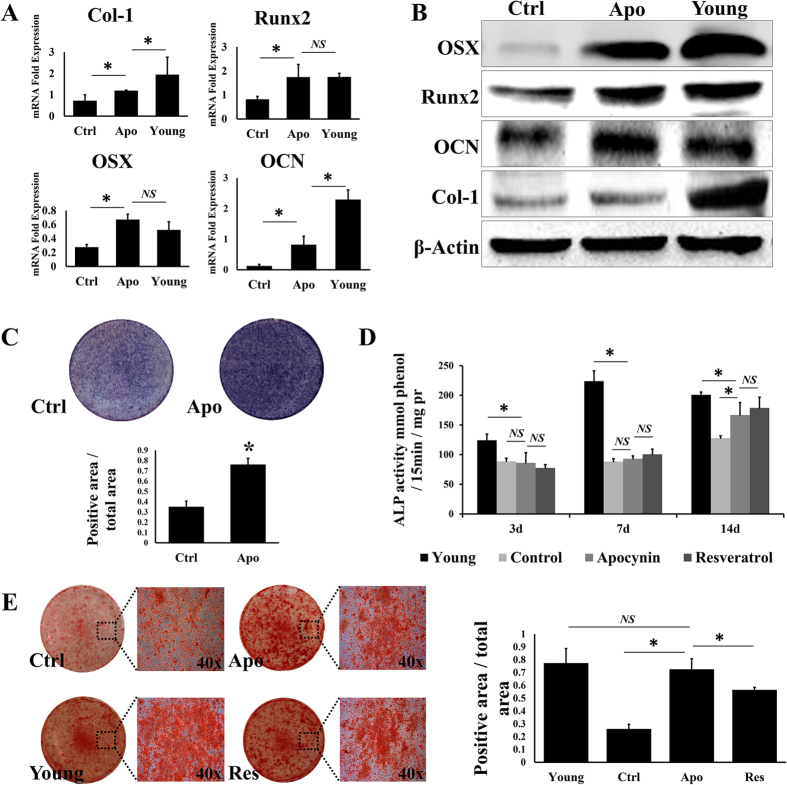
After osteogenic induction, the aging BMSCs treated with apocynin (100 μM) showed a significant increase in the expression level of an osteogenic marker (A,B). ALP staining and quantitative determination of ALP activity revealed that apocynin increased the expression of ALP compared with the negative control (**C**,**D**). The results of alizarin red staining showed that the percentage of mineralised nodules was increased by 3 fold under apocynin treatment, whereas it remained basically flat in the young cell group (**E**). All data are shown as the mean ± S.D.

We demonstrated that the suppression of NADPH oxidase using apocynin partially reversed the aging process in BMSCs. Next, we investigated whether this change affects osteogenesis in aging BMSCs. We compared the aging BMSCs treated with apocynin (100 μM) with the DMSO-treated negative control and young BMSCs isolated from 4-week-old SD rats. qRT-PCR data showed that the expression levels of four pivotal osteogenic markers (Runx2, OSX, Ocn, and Col1) were significantly higher under apocynin treatment (Fig. 4A). The expression levels of Runx2 and OSX (Osterix), which encode important transcriptional factors that regulate bone formation, were increased by 2 and 3 fold, respectively, on day 7 after osteogenic induction. Besides, the expression level of OSX was higher in the aging BMSCs treated with apocynin than in the young BMSC group, but not statistically significant. Ocn (osteocalcin) and Col1 levels were also significantly higher under apocynin treatment. The results of western blot analysis revealed similar trends in the levels of the proteins (Fig. 4B) and confirmed that the expression of the osteogenic markers in the aging BMSCs was enhanced under apocynin treatment.

Next, ALP (alkaline phosphatase) staining was conducted on day 14 after osteogenic induction in aging BMSCs treated with apocynin and the negative control cells. The results showed that apocynin (100 μM) significantly increased the percentage of ALP-positive cells, by 117% compared with the negative control (Fig. 4C). In the next experiment, we used resveratrol (50 μM) as a positive control because it was recently reported to greatly enhance osteogenesis in MSCs^22^,^23^. Quantitative analysis of ALP activity was conducted on days 3, 7 and 14 after osteogenic induction. Alizarin red staining was also performed on day 21 after osteogenic induction. The results of the quantitative analysis of ALP activity showed that there was no difference among the negative control, apocynin (100 μM) and resveratrol (50 μM) groups on days 3 and 7. However, on day 14, the apocynin group and resveratrol group showed much higher ALP activity than the negative control group, and their activity levels were similar to the level observed in the young BMSC group (Fig. 4D). There was no difference in the rate of alizarin red staining between the apocynin group and the young BMSC group, though the rate was slightly higher in the apocynin group compared with the resveratrol group. Both the apocynin and resveratrol groups showed a significant increase compared with the negative control group (2.8 and 2.2 fold, respectively) (Fig. 4E).

Ascorbic acid which was included in the osteogenic-inducing medium, is known to be an antioxidant^24^ that might cause changes of ROS level as well as apocynin. On the other hand, ascorbic acid plays a key role in osteogenic inducing^25^. To eliminate this interference factor, we treated the cells with ascorbic acid included/excluded osteogenic-inducing medium. The results showed that at day 3, cells treated with medium^asc-^ express significant lower col1a1 that cells treated with medium ^asc+^ (Fig. S5A). And at day 3 and day 7, the ALP activity was determined to be much less in cells treated with medium^asc-^(Fig. S5B). Cells treated with medium^asc-^ also showed a less rate of alizarin red staining at day 21(Fig. S5C). In all these results, apocynin was not able to enhance osteogenesis without the participation of ascorbic acid. Though the intracellular ROS level might be reduced, apocynin could not replace the role of ascorbic acid in osteogenesis.

Taken together, these results suggest that 100 μM apocynin significantly promotes the osteogenic potential of aging BMSCs. We attribute this increase in osteogenic potential to the apocynin-induced partial reversal of the aging process.

### Osteogenesis *in vivo*

To demonstrate the osteogenic potential of apocynin, we used a SAMP6 mouse model to evaluate the effect of apocynin *in vivo*. Male mice (n = 18) at 3 months of age were randomly divided into three groups (6/per group). Mice from the first group were executed and scanned via micro-CT and the other two groups were given further treatment. In one group, apocynin was administered 0.1 mg/Kg/day, through intraperitoneal injection three times per week. In the other group, normal saline was administered for comparison. After 3 months of injections, both groups of mice were euthanised, after which their shin bones were collected for micro-CT scanning, and the BMSCs were harvested and observed.

**Figure 5:**
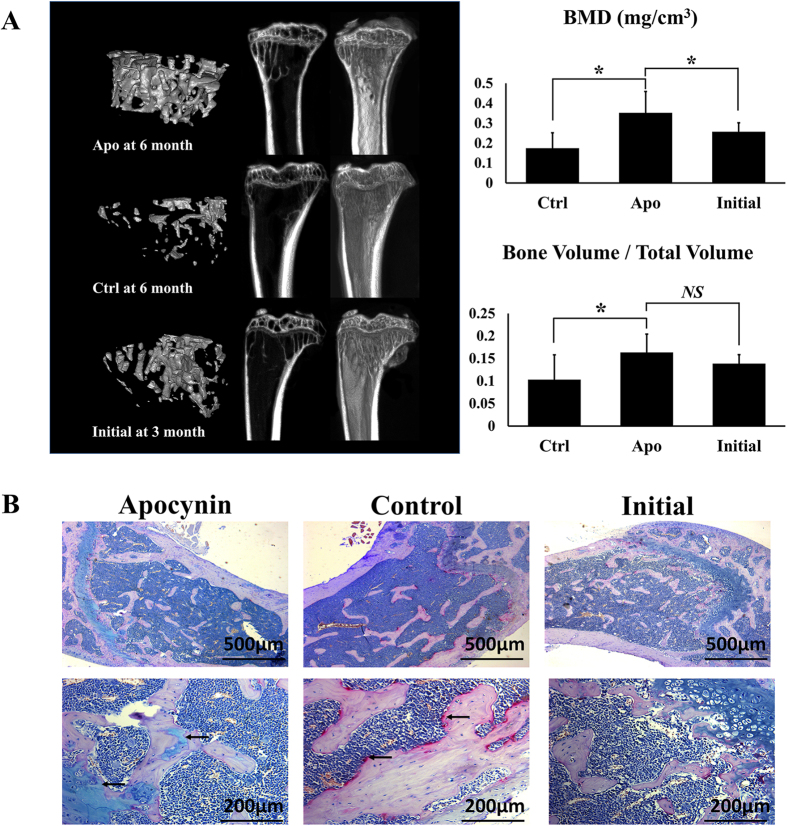
The bone was traced via micro-CT before and after drug treatment. Images from CT scans and restitution were collected at 3 and 6 months; Initial: SAMP6 mouse model at 3 months without drug treatment (n = 6); Apo: 3 months old mice subjected to 3 months of intraperitoneal injections with apocynin (0.1 mg/Kg/day, given 3 days per week, n = 6); Ctrl: Mice subjected to 3 months of intraperitoneal injections with normal saline, used as a negative control group (n = 6). The quantification of BV/TV and BMD in the shin bones was performed by CT (B, BV/TV; C, BMD). The Apo group showed increased BMD and BV/TV values compared with the Ctrl group. All data are shown as the mean ± S.D. (**A**). ALP –TRAP staining was performed. The blue area inside the trabecular bone indicates the activity of osteoblasts, and the red region at the margin of the trabecular bone represents the activity of the osteoclasts. The Apo group showed a noticeable increase in osteoblast activity, and in the Ctrl group, the osteoclasts were more active (**B**).

The micro-CT results showed that after 3 months of apocynin treatment, the BMD (bone mineral density) value was significantly higher compared with the negative control group (0.352 mg/cm^3^ vs. 0.152 mg/cm^3^). Compared with the initial value measured at 3 months (0.258 mg/cm^3^), the BMD value in the apocynin group was increased by 1.36 fold. Conversely, the BMD value in the control group was reduced by 41%. The trend in the BV/TV (bone value/total value) was similar to the BMD value. The apocynin group showed a higher bone value than the control group (Fig. 5A).

BMSCs from both the apocynin group and control group were isolated from the thigh bones, and SA-β-gal staining was conducted. The results showed that in the primary cell passage and passage 2, the two groups presented no significant differences, while in passages 3 and 4, the apocynin group exhibited a lower percentage of SA-β-gal positive cells than the control group (Fig. S4A). The obtained cell number counts revealed that on day 7, the number of cells in the apocynin group was 1.28-fold higher than in the control group (Fig. S4B). Level of Ki67 was also detected by cell immunofluorescence and results showed that apocynin group express more Ki67 than the control group (Fig. S4C). The level of intracellular ROS was determined, and the results revealed that apocynin injections reduced the level of intracellular ROS in the BMSCs (Fig. S4D). RT-PCR results showed that the p53 mRNA expression level was lower, while Oct-4 mRNA expression was higher in the apocynin group compared with the control group (Fig. S4E). Osteogenic induction was also performed, and ALP activity was determined on day 3 and day 7. The apocynin group showed more activity in this quantitative analysis (Fig. S4F).

After the micro-CT analysis, the shin bones of the animals from both groups were decalcified, and ALP-TRAP staining was performed. The results showed that apocynin altered the osteoblast-osteoclast balance in bone. The trabecular bone volume was lower in the initial group than in the 6-month group. Compared with the initial time point, 3 months of apocynin treatments substantially promoted the activity of osteoblasts, while in the negative control group, we observed very little osteogenic activity, and osteoclasts were active by a large margin (Fig. 5B).

## Discussion

Cellular senescence is closely associated with an organism’s aging and longevity. Although many studies have reported that increased antioxidant defences do not extend the lifespan^26^,^27^,^28^, the relationship between oxidative stress and cellular senescence is strongly supported^29^,^30^. Accelerated senescence in bone marrow stromal cells has been shown to be one of the key factors in bone aging^6^, and oxidative stress undoubtedly affects the aging process of BMSCs. There are various downstream targets of ROS related to aging^31^, which control cell metabolism through different pathways. Among all these targets, p53 has received significant attention because it is at the centre of various pathways and has a major impact on cell senescence^10^,^32^. How the interaction between ROS and p53 regulates aging remains unclear, but it is generally accepted that multiple effects occur during the ROS/p53-mediated aging process^33^. In this study, we first demonstrate that in BMSCs, down-regulation of intracellular ROS suppresses the expression of p53 by ATM signaling, which results in a partial reversal of the aging process and leads to an increased potential for osteogenesis. ATM was directly response to DNA damage^34^ caused by ROS. And with the active of ATM, p53 was activated and leading to a series of changes in aging process.

There are many factors that are responsible for the generation of intracellular ROS, such as mitochondrial respiratory chain, xanthine oxidase, cytochrome P450 and NADPH oxidase. And among all these factors, NADPH oxidase is able to stimulate one or more factors above to generate more ROS^35^. It is generally believed that NADPH oxidase is the source of ROS. And the NADPH oxidase inhibitor, apocynin, can effectively reduce the ROS level by inhibiting the activity of NADPH oxidase^15^.

Nanog and Oct-4 are vital transcription factors in the self-renewal of stem cells^20^,^21^, and the expression of Nanog and Oct-4 decreases as a cell ages^36^,^37^. Therefore, we choose Nanog and Oct-4 as the targets of a screen of apocynin concentrations; an increase in Nanog and Oct-4 expression indicates reversal of the aging process, or at least a partial reversal. Hence, Nanog and Oct-4 were selected as the targets of the screen of apocynin concentrations to ensure that the NOX inhibitor was used at the optimum concentration for regulating the aging process in BMSCs.

The mRNA and protein expression levels of p21 were suppressed by apocynin, and we speculate that reduced expression of p53 was responsible for this suppression^38^. In contrast, the cell cycle and cell proliferation did not appear to be altered in any way. This finding indicates that the down-regulation of p21 alone is not sufficient to alter cell cycle arrest or to improve proliferation in aging BMSCs^39^. The increased expression of klf-4 and sox-2 demonstrated that the potential for self-renewal in the aging BMSCs was improved, which is consistent with the observed expression levels of Nanog and Oct-4. The expression levels of ap2 and c-myc were higher, and this finding suggests that the differentiation potential of the aging BMSCs was improved^40^,^41^. The data also showed that the TERT protein expression level was increased, whereas the TERT mRNA expression level was not. This finding indicates that the increase in TERT expression occurs at the translational level. Additionally, we sought to determine whether these aging-associated changes were linked to the SIRT family, which is crucial to the aging process^8^,^42^,^43^. The results showed that sirt1 and sirt2 mRNA expression levels did not were not altered. This finding indicates that in aging BMSCs, SIRT may not be involved in the reversal of the aging process caused by intracellular ROS suppression.

With the increased expression of “stemness markers”, we speculated that osteogenesis in aging BMSCs should be improved^44^,^45^, and our results demonstrated that the osteogenic potential of aging BMSCs was clearly enhanced under apocynin (100 μM) treatment. This finding indicates a positive evaluation in aged bone formation. Furthermore, *in vivo* data support this phenomenon. With continuous apocynin (100 μM) treatment, the shin bones of the SAMP6 mice showed a higher BMD value and a greater bone volume compared with the control group. In our previous study, another small molecular compound Licochalcone A was used to treat SAMP6 mice^46^. And our results showed that a 3 month treatment is appropriate to find whether the compound is effective for the mice. So a 3 month treatment was also used to determine the effect of apocynin. The data also showed that after 3 months of apocynin injections, the BMSCs isolated from the thigh bone presented a lower intracellular ROS level and more activity in terms of proliferation and osteogenic differentiation. Furthermore, the activity of the osteoblasts was promoted, while osteoclasts were suppressed. These findings may explain the greater amount of bone formation observed in the apocynin group.

Besides these work, there are still some questions need to be solved. We attempted to explain how apocynin affects the aging process in BMSCs, but our work was not sufficient. We demonstrated that apocynin regulates ROS levels in BMSCs, which is related to the expression of p53. However, the mechanism underlying this relationship remains unclear. Currently, we know that the aging process mediated by ROS and p53 is complex and that there are various interactions between ROS and p53^47^. Moreover, whether the change in p53 expression is the primary factor responsible for the reversal of the aging process and the initiation of the enhanced osteogenesis potential was only preliminary explored in this study. Besides, RT-PCR results showed that expression of AP2 was increased by the effect of apocynin. So the potential of adipogenesis of aging-BMSCs might also be changed, and this need further study to identify how BMSC react to this phenomenon. Additionally, the BMSCs isolated from SD rats and SAMP6 mice showed differences in proliferation. Apocynin treatment had no effect on proliferation in the BMSCs from the SD rats, whereas it promoted proliferation in the BMSCs from the SMP6 mice. We speculate that the different methods of drug administration are responsible for these different results. In addition, Bone marrow cavity is a hypoxic condition, and in this condition, BMSCs represent a great difference comparing to BMSCs in the normoxia conditions^48^. Many studies have found that different oxygen tension causes different changes of BMSCs in proliferation, migration or differentiation^49^,^50^,^51^. And to investigate how BMSCs react to apocynin in the marrow cavity, to build a hypoxic conditions is of more significance. We will explore the mechanism of aging-BMSCs reacting to apocynin in hypoxic condition in our further study.

In conclusion, we demonstrated that in an aging mesenchymal stem cell model, oxidative stress is closely associated with the aging process. We investigated the changes in cell behaviour of aging BMSCs stimulated with apocynin and found that at a 100 μM concentration, apocynin partially reversed the aging process in BMSCs. The aging BMSCs showed enhanced self-renewal and a significant increase in osteogenic potential with the apocynin treatment. We demonstrated that apocynin suppresses the expression of NOX and results in a reduced intracellular ROS level. The decreased expression of p53 resulting from the reduced ROS level is important to the reversal of the aging process in BMSCs. The results also showed that in the SAMP6 mouse model of premature aging, apocynin significantly increased the BMD value and bone volume by affecting the BMSCs. The potential for proliferation and osteogenesis was promoted in the BMSCs, which is the primary reason for the increase in bone formation.

## Methods

### Animals

SD male rats were obtained from the Laboratory Animal Research Centre of the Forth Military Medical University. BMSCs from 22-month-old and 4-week-old SD rats were used for culture. All animal experiments were conducted in accordance with the committee guidelines of the Fourth Military Medical University, Xi’ an, China and met the NIH guidelines for the care and use of laboratory animals. Animal protocols were approved by the IACUC committee of FMMU at Xi’an.

### Culture of rat BMSCs

Primary SD rat BMSCs were isolated as previously described^52^. Briefly, BMSCs were obtained from 22-month-old and 4-week-old rats and were used as models of aging BMSCs and young BMSCs, respectively. Cells from passages 3–5 were used in the experiments. Apocynin was purchased from Merck (Merck Co., USA) and was dissolved in DMSO to obtain a 2 mM solution and was used at final concentration of 100 μM unless otherwise indicated.

### Osteogenic differentiation

A total of 1 × 10^5^ BMSCs were seeded into each well of a 6-well plate (Nunc). Apocynin was added before the induction of osteogenic differentiation. At 80% confluence, apocynin was removed and the BMSCs were cultured in osteogenic-inducing medium containing 10% FBS, 5 mM L-glycerophosphate, 100 nM dexamethasone (Sigma), and 50 mg/ml ascorbic acid for a certain number of days. The osteoblast phenotype was evaluated by determining the expression levels of marker genes via qRT-PCR and alizarin red staining using 40 mM alizarin red S (ARS; Sigma-Aldrich).

### Assessment of senescence-associated β-galactosidase (SA-β-gal) activity

SA-β-gal was visualised using X-gal (5-bromo-4-chloro-3-indolyl-β-D-galactopyranoside) (Beyotime, China) (Stenderup *et al.*, 2003). SA-β-gal–positive cells were green/blue. The stained cells were photographed under an optical inverted microscope (Olympus cx31) and evaluated using Image-Pro Plus software (version 7.0).

### Cell-cycle analysis

BMSCs were trypsinised, washed with PBS, suspended in 50 μg/ml propidium iodide in 0.1% (w/v) trisodium citrate dehydrate and 0.1% (v/v) Triton X-100 in PBS and incubated for at least 1 h at 4 °C. The BMSCs were then analysed via flow cytometry using the FL-2A channel (FACSCalibur; BD Biosciences). The percentages of cells in different phases of the cell cycle were calculated using CellQuest Pro software (BD Biosciences).

### Cell proliferation assay

Cell proliferation was assessed using the MTT assay. BMSCs were plated into 96-well tissue culture plates at 3*10^3^ cells/well. The cells were allowed to grow for 7 days. Every 24 h, the medium was changed to serum-free medium, and 10 ml of 0.5 mg/mL MTT was added to each well, followed by incubation for another 4 h. The medium was then discarded, and formazan salts were dissolved in 100 μL of DMSO, after which the plate was read at 570 nm using a microplate reader (Bio-Rad, Model 550)^53^.

### Quantitative real-time PCR

Total RNA was isolated using the TRIzol reagent (Invitrogen) according to the manufacturer’s standard instructions. For reverse transcription of mRNA, random-primed cDNA was synthesised from 2 mg of total RNA using the PrimeScript RT reagent kit (TaKaRa, Dalian, China). Real-time PCR was performed using 2 μL of the cDNA product in a 25 μL reaction volume on a 7500 Real Time PCR System (Applied Biosystems, Singapore). SYBR Premix Ex TaqTM II (Takara Biotechnology), specific primers (see below) and 2 μL of cDNA were used in each amplification reaction (95 °C for 30 s, 40 cycles of denaturation at 94 °C for 5 s and annealing and extension at 60 °C for 30 s). Sense and antisense primers were designed based on published cDNA sequences using Primer Express 5.0. Actin was used as an internal control gene. The primer sequences are listed in Table 1. All of the real-time PCR assays were performed in triplicate, and the results obtained after calibration based on the ACTIN expression level were calculated using the ^ΔΔ^CT method and are presented as the fold increase relative to the non-stimulated control.

### Western blot analysis

The protein levels of Runx2, Ocn, OSX, Col1, p53, p-p53, ATM, p-ATM, p21, p16 and TERT in aging BMSCs were assessed using western blot analysis. Protein concentrations were determined via Bradford protein assays. Protein (100 μg) from each sample was loaded onto a 7.5% polyacrylamide gel. After electrophoresis, the proteins were transferred to a PVDF membrane (Millipore). The membrane was then blocked with 5% nonfat milk in PBS and incubated with anti-Runx2, anti-Ocn, anti-OSX, anti-TERT (Santa Cruz), anti-col-1a1 (epitomics), anti-p53 (Cell Signaling), anti-p16INK4 (Abbiotec), anti-p21, or anti-β-actin (Abcam, USA) in PBS for 3 h. The membrane was subsequently re-probed with the appropriate secondary antibody conjugated with horseradish peroxidase for 1 h. The blots were processed using an ECL kit (Santa Cruz Biotechnology) and exposed to film. The protein levels were normalized by β-actin.

### Cell Immunofluorescence

Expression of Ki67 was detected by cell immunofluorescence in aging-BMSCs treated with or without apocynin. After harvest, cells were fixed in 4% paraformaldehyde and then treated with 0.5% triton X-100 (Sigma-Aldrich, US) for 20 min. After blocking with 5% goat serum (Millipore, US), samples were incubated in Ki67 primary antibody (Abcam, US) overnight at 4 °C. On the next day, samples were treated with Alexa Fluor 594 affinipure donkey anti-rabbit IgG (Jackson, US) and the 0.1 mg/ml of 40, 6-diamidino-2-phenylindole (DAPI, Sigma-Aldrich, US) was used to stain the nuclear. All samples were observed under a fluorescence microscope (FV1000, Olympus, Japan) and the average optical density was calculated by Image Pro Plus 6.0.

### DNA Damage Quantification

DNAiso Reagent (Takara Bio, Japan) was used to extract total DNA. After extraction, DNA Damage Quantification Colorimetric Kit (#K253-25, Bio Vision) was used to detect DNA damage quantification. All operation was performed according to the manufacturer’s instructions. Briefly, the Kit utilizes the ARP (Aldehyde Reactive Probe) reagent that reacts specifically with an aldehyde group which is the open ring form of the AP sites. After treating DNA containing AP sites with ARP reagents, AP sites are tagged with biotin residues, which can be quantified using avidin-biotin assay followed by a colorimetric detection.

### Assessment of ALP staining and ALP activity

The osteogenic differentiation potential of the BMSCs was evaluated through ALP staining. ALP activity was measured using a commercial kit according to the manufacturer’s instructions (Nanjing Jiancheng Bioengineering Ltd., Nanjing, China). The numbers of colonies positive for ALP staining on day 14 were also compared.

### Assessment of NADPH oxidase activity

A kit for the quantitative detection of cell NADPH oxidase activity via colourimetry (Genmed, Shanghai) was used to determine the activity of NADPH oxidase in BMSCs. A total of 5*10^6^ aging BMSCs from each sample were trypsinised and washed with PBS, and total protein was then extracted. Protein concentrations were determined using the Bradford protein assay. The NADPH oxidase activity in each sample was measured using an enzyme micro-plate reader (Biotec) according to the manufacturer’s instructions (Genmed, GMS50096.1). The absorbance was measured at 550 nm. The total activity and the non-specific activity were measured. The specific activity of each sample was calculated and plotted as a histogram. The values are expressed as μmol NADPH/min/mg.

### Measurement of reactive oxygen species (ROS)

A kit for the detection of intracellular reactive oxygen species based on primary fluorescence (Genmed, Shanghai) was used to detect intracellular ROS levels in BMSCs. The oxidation-sensitive fluorescent probe DCFH-DA was employed to monitor the production of intracellular ROS. For quantitative detection, a total of 5*10^6^ aging BMSCs from each sample were trypsinised and washed with PBS. The cells were then incubated for 20 min at 37 °C in the presence of 10 μmol/L DCFH-DA, with gentle agitation. Next, the reaction was stopped, and the fluorescence intensity was measured via flow cytometry using the FL-2A channel (FACSCalibur; BD Biosciences) with excitation at 490 nm and emission at 530 nm. At least 50,000 cells were counted for each sample. Cellular fluorescence was quantitated using the geometric means of the data distributions. For qualitative detection, aging BMSCs from each sample were seeded into a glass-bottom plate (Nunc), after which DCFH-DA was added (final concentration, 10 μmol/L), and the cells were incubated for 20 min at 37 °C in a CO_2_ incubator. Then, the reaction was stopped, and fluorescence was measured using a Zeiss LSM 510 laser-scanning confocal microscope (Gottingen, Germany).

### Micro-CT and histological analysis

Micro-CT was used to scan the shin bones of SAMP6 mice at a voltage of 80 keV with a current of 500 mA. After automatic reconstruction, 2D slices with a 19 mm isotropic resolution were generated and used for 3D reconstruction. For the initial analysis, a square was internally tangent to the defect circle in the selected region of interest. After choosing a determinate threshold, the new bone volume fraction (BV/TV) and BMD were calculated using built-in software. Mouse shin bones were collected before and after drug treatment at 3 months and 6 months, respectively. Then, the shin bones were fixed in 10% neutral-buffered formalin, transferred to 70% ethanol and finally decalcified in 9% formic acid. After this processing, the samples were embedded in paraffin. Sagittal sections (4 mm) were cut along the cranial shaft axis, collected on glass slides, deparaffinised and subjected to haematoxylin-eosin (H&E) and ALP-TRAP staining. After mounting with cover slips, the specimens were viewed and analysed under a light microscope (Olympus, Japan). The total area of newly formed bone tissue and newly formed bone tissue around the CBB were measured using Image-Pro Plus 6.0 software.

### Statistical analysis

All experiments were repeated at least three times, and the data are presented as the mean ± SD. Statistical significance was analysed using SPSS 11.0 software (SPSS). P < 0.05 was considered statistically significant.

## Additional Information

**How to cite this article**: Sun, J. *et al.* Apocynin suppression of NADPH oxidase reverses the aging process in mesenchymal stem cells to promote osteogenesis and increase bone mass. *Sci. Rep.* **5**, 18572; doi: 10.1038/srep18572 (2015).

## Supplementary Material

Supplementary Data
