# Supplementary material for: Apocynin suppression of NADPH oxidase reverses the aging process in mesenchymal stem cells to promote osteogenesis and increase bone mass
Source: Sci Rep. 2015 Dec 21;5:18572. doi: 10.1038/srep18572 (PMC4685263; doi:10.1038/srep18572)
Supplement: Supplementary Data [file srep18572-s1.pdf]

Apocynin suppression of NADPH oxidase reverses the aging process in mesenchymal stem cells to promote osteogenesis and increase bone mass

Jinlong Sun[1,2,4,a], Leiguo Ming[2,3,5,a], Fengqing Shang[2], Lijuan Shen[4], Jihua Chen[4,*], Yan Jin[1,2,3,5,]**

[1] State Key Laboratory of Military Stomatology, Center for Tissue Engineering, School of Stomatology, The Fourth Military Medical University, Xi'an, Shaanxi 710032, China.

[2] Research and Development Center for Tissue Engineering, Fourth Military Medical University, Xi'an, Shaanxi 710032, China.

[3] State Key Laboratory of Military Stomatology, Department of Oral Histology and Pathology, School of Stomatology, Fourth Military Medical University, Xi'an, Shaanxi 710032, China.

[4] State Key Laboratory of Military Stomatology, Department of Prosthodontics, School of Stomatology, Fourth Military Medical University, Xi' an, Shaanxi 710032, PR China

[5] Institute for Tissue Engineering and Regenerative Medicine Research of Xi'an, Xi'an, Shaanxi, 710032, China

* Corresponding author. 145 West Changle Road, Xi'an, Shaanxi 710032, PR China. Tel.: +86-029-84776329; jhchen@fmmu.edu.cn (J. Chen).

** Corresponding author. 145 West Changle Road, Xi'an, Shaanxi 710032, PR China. Tel.: +86-029-84776147; fax: +86-029-83218039; yanjinfmmu@139.com (Y. Jin),

[a] Shared first co-authorship as both authors contributed equally to this work.

karmailsun@hotmail.com (J. Sun)

Supplemental Table:

Primer	Sequences
r-nanog-F	TCCTCACCAAGAAAGCAGAAGAT
r-nanog-R	GCTCAGGCTCAGAATGGTAGAGA
r-oct4-F	TCTACTCGGTCCCTTTTCCTGA
r-oct4-R	TTTGTCTACCTCCCTTCCTTGC
r-P53-F	CAGATTGGGGAATGGGTTGG
r-P53-R	GCAGAGTGGAGGAAATGGGTC
r-Glb-F	TTTCTGGGGACCGTGATGTG
r-Glb-R	CTCTAGTAACCAAGCGGGTAAGC
r-P21-F	CACAGGAGCAAAGTATGCCGTC
r-P21-R	GCGAAGTCAAAGTTCCACCGT
r-Osterix-F	GCCTACTTACCCGTCTGACTTT
r-Osterix-R	GCCCACTATTGCCAACTGC
r-OCN-F	ATGAGAGCCCTCACA
r-OCN-R	AGAGCGACACCCTAGAC
r-actin-F	TCCTGCGTCTGGACCTGG
r-actin-R	ACCGCTCATTGCCGATAGTG
r-P16-F	TCGTGCGGTATTTGCGGTAT
r-P16-R	CCAGAAGTGAAGCCAAGGAGAA
r-Col-1-F	TTCCCGGTGAATTCGGTCTC
r-Col-1-R	ACCTCGGATTCCAATAGGACCAG
r-Runx-2-F	GCACCCAGCCCATAATAGA
r-Runx-2-R	TTGGAGCAAGGAGAACCC
r-tert-F	TCAGTCTTGCGGTTGAAGTGTC
r-tert-R	GCCAAAGGGAAGCCGAATC
r-Sirt1-F	CATAGGTTAGGTGGCGAGTATGC
r-Sirt1-R	AAATATGAAGAGGTGTTGGTGGC
r-Sirt2-F	CCTGTGGAAAAGAGTACACGATG
r-Sirt2-R	GCTAGTGGTGCCTTGCTGATG
r-Klf4-F	CGGTCATCAGTGTTAGCAAAGG
r-Klf4-R	TCGGGACTCAGTGTAGGGGTA
r-Sox2-F	CATGACCAGCTCGCAGACC
r-Sox2-R	GCCTCGGACTTGACCACAGA
r-c-myc-F	CCTACATCCTGTCCGTTCAAGC
r-c-myc-R	CAACTGTTCTCGCCGTTTCCT
r- aP2-F	AGAAGGGGACTTGGTCGTCA
r- aP2-R	TCCACGCCCAGTTTGAAGG

Table1： Primer sequences used in this research

Supplemental Figures:

Figure S1:

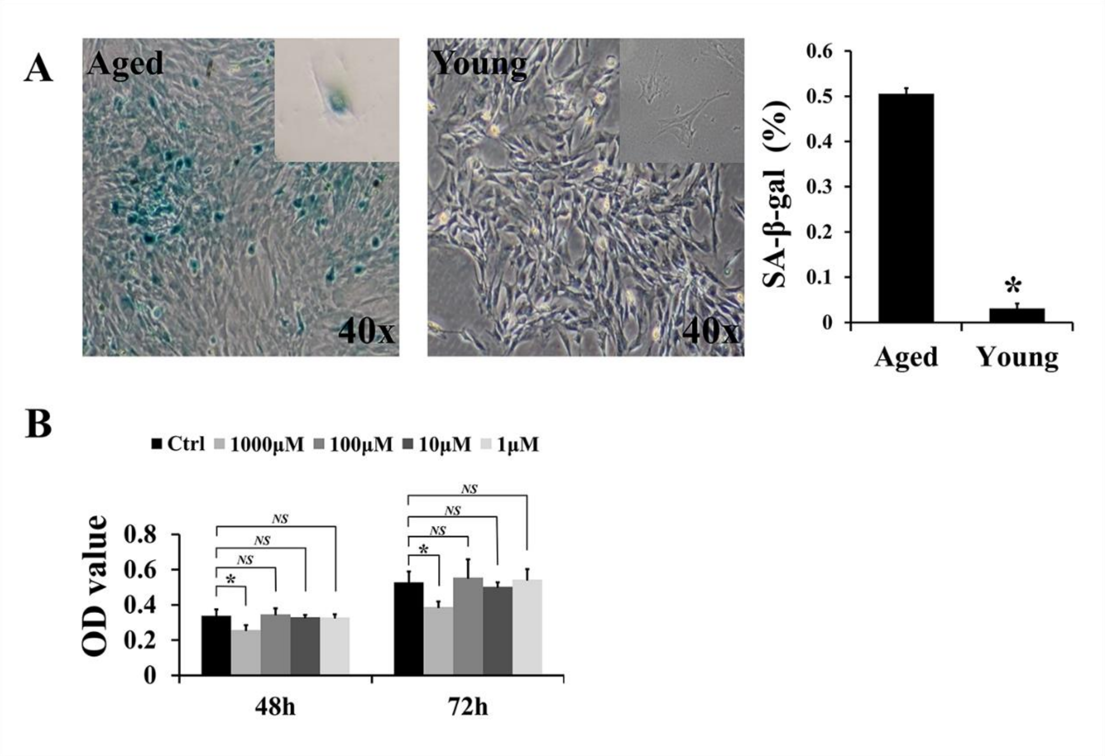

Fig S1. BMSCs isolated from 22-month-old and 4-week-old SD rats were stained with SA-β-gal, and the recorded percentages of stained cells were 50.55±1.22% and 3.10±1.05%, respectively (A). The MTT assay was used to determine whether apocynin at different concentrations suppressed cell proliferation (B).

Figure S2:

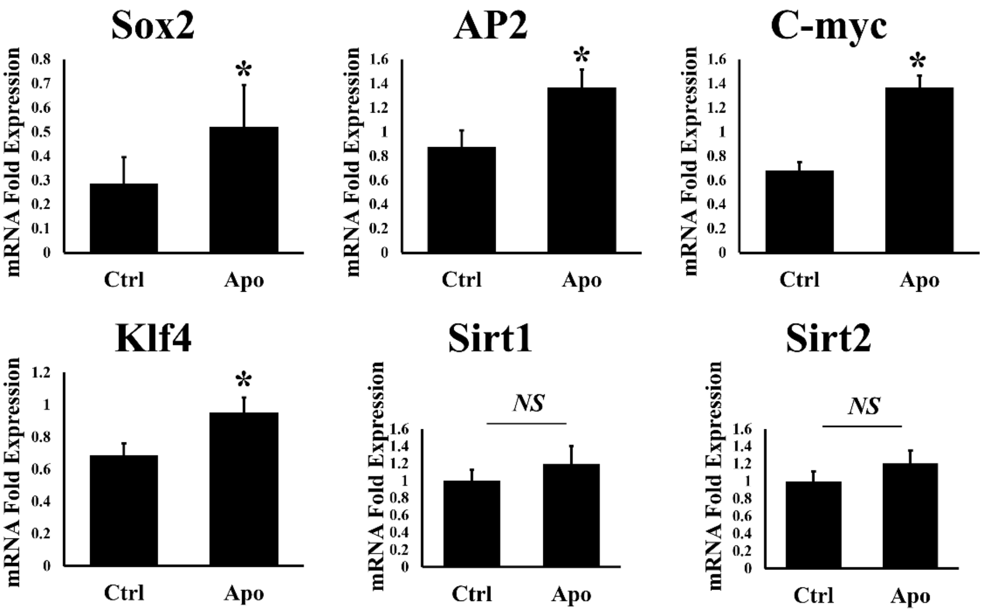

Fig S2. RT-PCR results showed that the expression of the age-related targets AP2 and c-myc was increased, as were sox-2 and klf-4 expression levels under apocynin treatment. Apocynin did not change the expression of sirt1 and sirt2.

Figure S3:

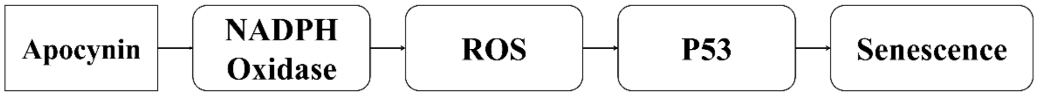

Fig S3. Speculation regarding how apocynin influences the aging process in BMSCs. We speculated that apocynin down-regulated the activity of NADPH oxidase in aging BMSCs, which decreased the level of intracellular ROS, leading to a series of changes in downstream targets, particularly in the expression of p53. The suppression of p53 activated downstream targets and resulted in aging-associated changes.

Figure S4:

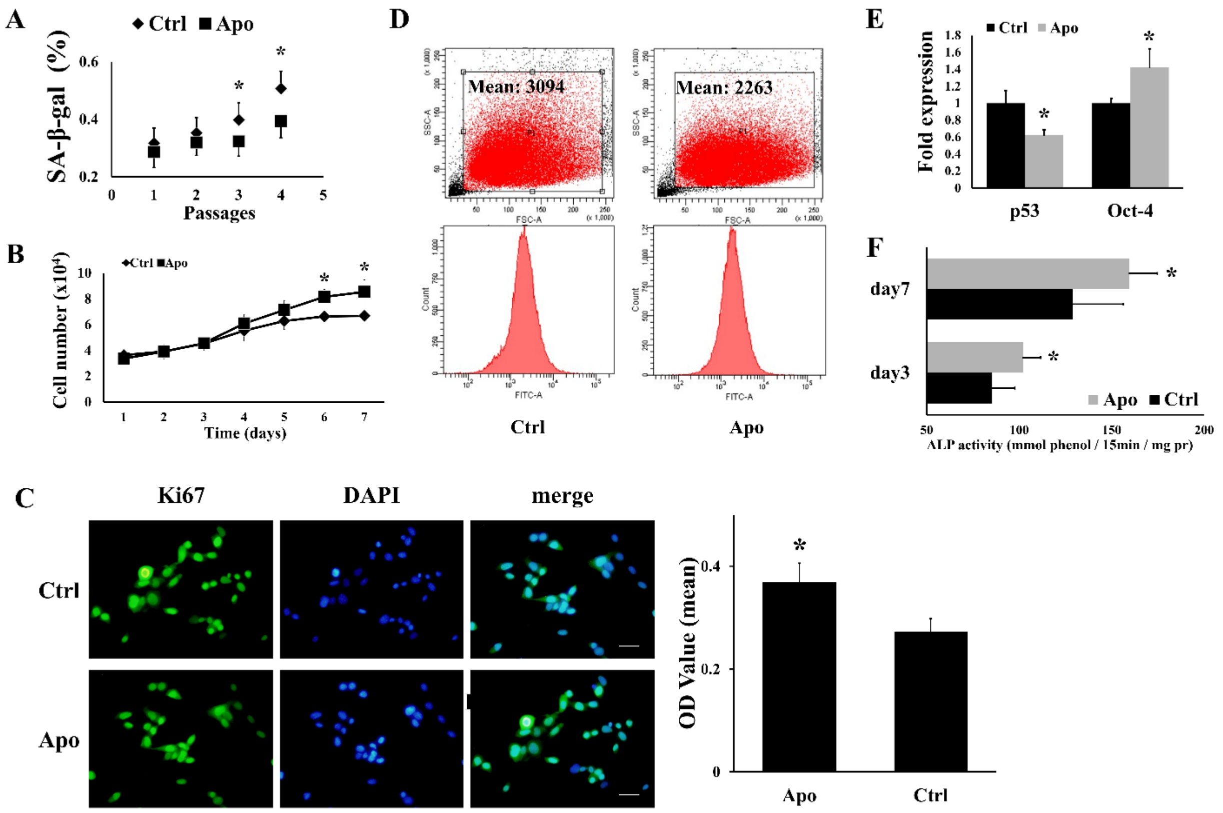

Fig S4. At 6 months, BMSCs isolated from the thigh bones of SAMP6 mice were cultured and tested. SA-β-gal staining and cell proliferation assays were performed. The Apo group showed lower SA-β-gal activity at passages 3 and 4 （A） and an enhanced proliferative potential (B&C). The Apo group also showed a lower intracellular ROS level (D). The PCR results showed that p53 expression was suppressed and oct-4 expression was enhanced (E). After osteogenic induction, ALP activity in the Apo group was also enhanced (F).

Figure S5:

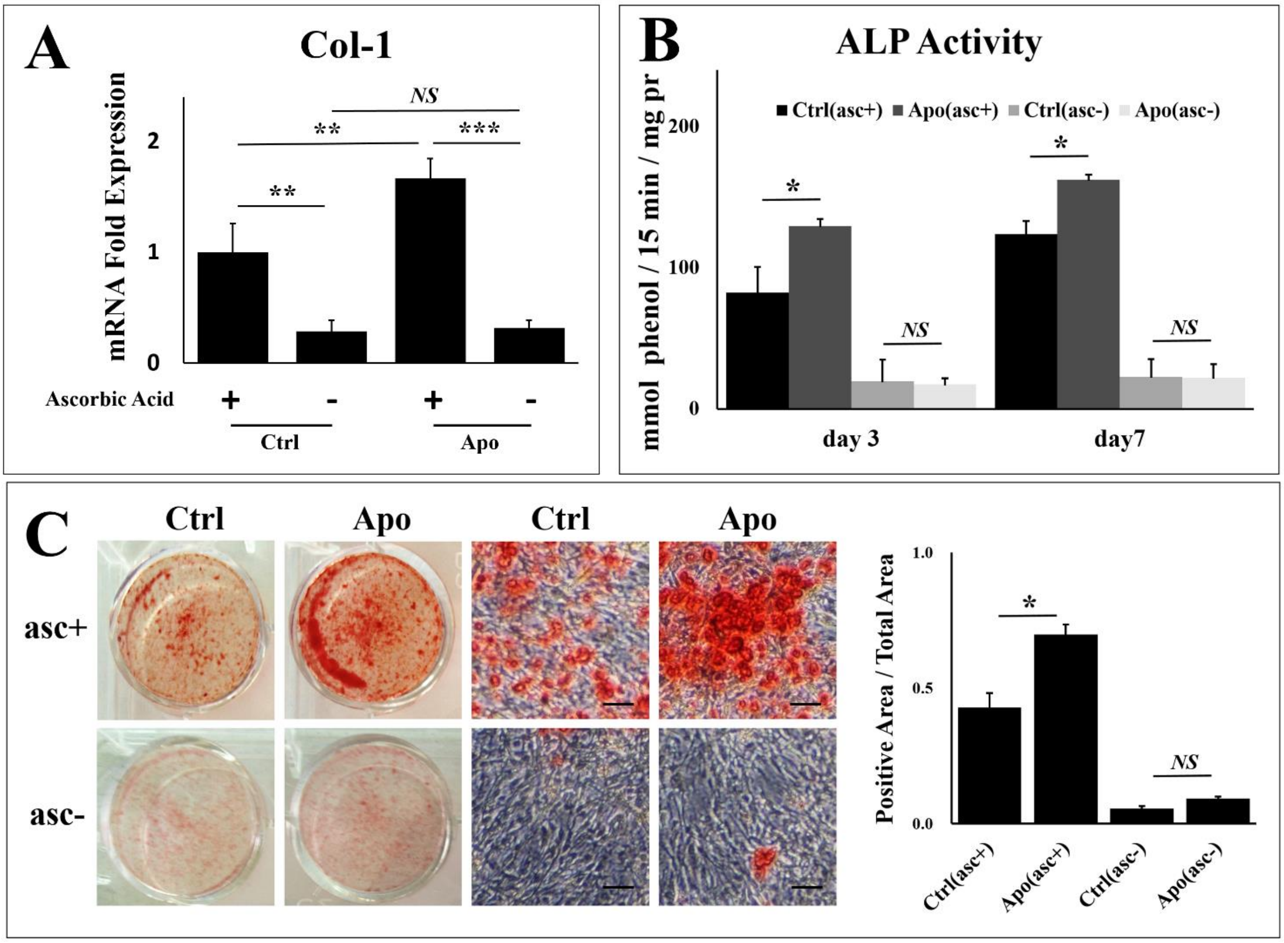

Fig S5. Aging-BMSCs were treated with ascorbic acid included/excluded osteogenic-inducing medium. The results showed that at day 3, cells treated with medium^{asc-} express significant lower col1a1 that cells treated with medium^{asc+} (A). And at day 3 and day 7, the ALP activity was determined to be much less in cells treated with medium^{asc-}(B). Cells treated with medium^{asc-} also showed a less rate of alizarin red staining at day 21(C). In all these results, apocynin was not able to enhance osteogenesis without the participation of ascorbic acid.
